# Patient Safety in Surgery: practical tips for authors to circumvent the journal’s high rejection rate

**DOI:** 10.1186/s13037-020-00267-1

**Published:** 2020-10-21

**Authors:** Philip F. Stahel, Sebastian Weckbach

**Affiliations:** 1 Department of Specialty Medicine, College of Osteopathic Medicine, Rocky Vista University, 8401 S. Chambers Rd., Parker, CO 80134 USA; 2 NeuroSpineZürich, Seestrasse 315, CH-8038 Zürich, Switzerland

**Keywords:** Patient safety in surgery, Submission guidelines, Rejection rate, Tips for authors

The open-access peer-reviewed journal *Patient Safety in Surgery* is currently in its 14th year of publication, with a growing readership around the globe [1]. The journal’s articles have been read more than 1.5 million times until present. In the past 5 years, *Patient Safety in Surgery* published the work of more than 500 researchers from around 200 different institutions. Authors from more than 50 countries have published in the journal (Fig. 1). The top-10 countries with the highest number of publications are shown in Table 1. With the increasing number of submissions over the years, the scrutiny around the rigorous acceptance standards have improved in parallel. The journal has a current rejection rate of 50% for all submissions. The main reason for the increasing rejection rate is the editorial board’s intent of assuring novel, hypothesis-driven, high-quality research with scientific merit and clinical relevance pertinent to the field of surgical patient safety.

**Fig. 1:**
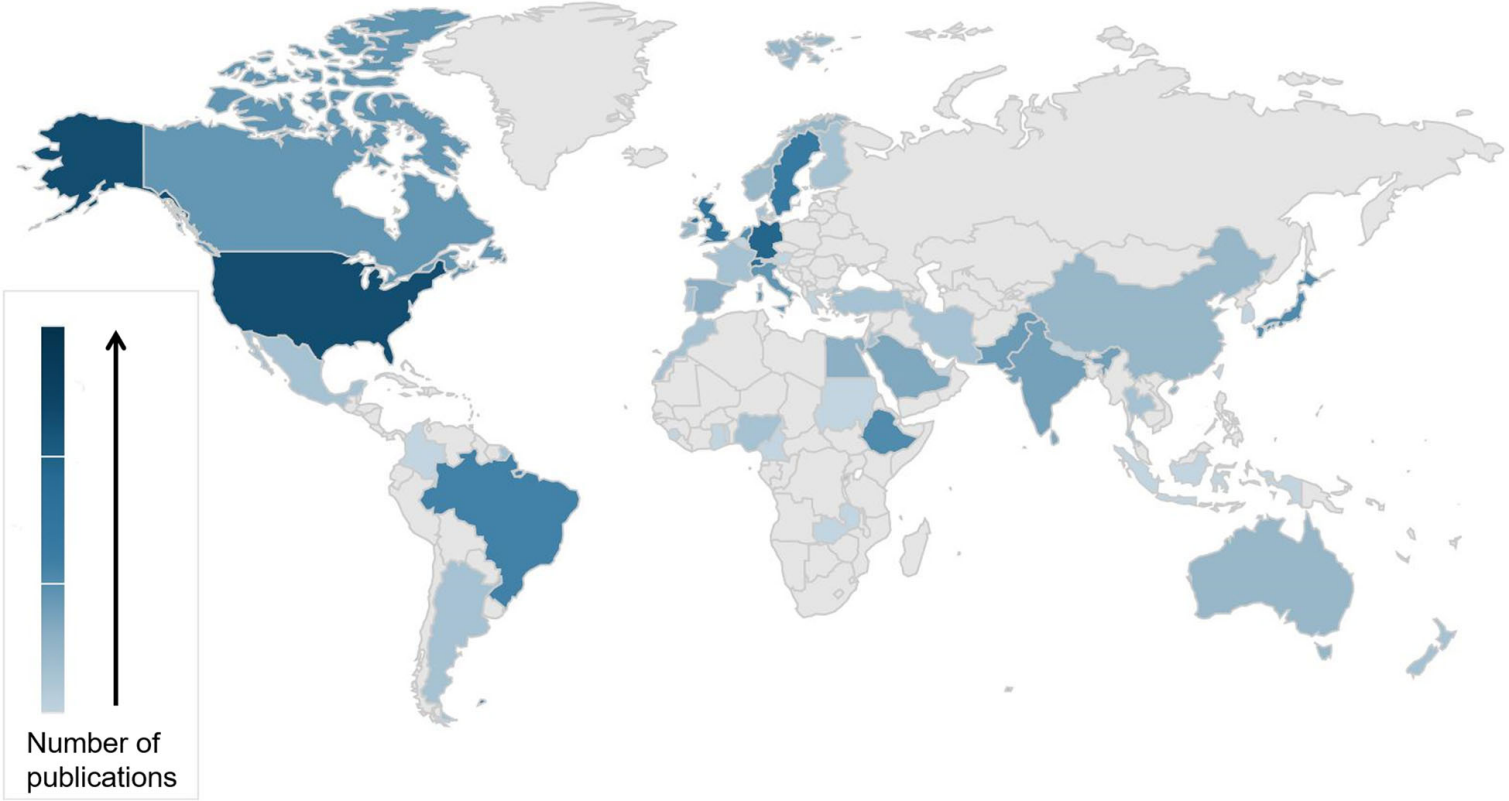
Countries of origin for publications in *Patient Safety in Surgery*

**Table 1:** Top-10 countries with the highest number of publications in the journal

Country	Rank	No. of publications^a^
USA	1	190
Germany	2	82
Switzerland	3	52
United Kingdom	4	35
Sweden	5	21
Brasil	6	14
Ethiopia	7	11
Japan	7	11
Netherlands	9	10
Canada	10	8

^a^ as of September 1, 2020

Manuscripts deemed of appropriate scientific quality are sent out for peer-review by at least two referees. However, many submissions are rejected at the initial stage of in-house editorial board screening (“reject without review”) largely due to poor quality presentation and content. Most of these concerns are preventable by applying due diligence to article formatting and proof-reading prior to submission. Some of the frequently identified concerns during the initial screening of rejected manuscripts include the following:

- Poor presentation with spelling errors, inconsistencies in formatting, and substandard scientific terminology and English language.
- Incoherent study design/methodology and frequent absence of a study hypothesis.
- Hypothetical extrapolation of conclusions beyond the objective data shown.
- Alleged statistical significance (based on *P*-values < 0.05) without apparent clinical relevance (“so what?” question by referees).
- Manuscript not formatted according to the specific instructions for authors [2].
- Citations incomplete or not formatted according to the journal’s style.

In essence, the notion that “sloppy presentation implies sloppy science” will negatively impact the referees’ evaluation of a submitted manuscript, independent of the quality of the underlying data. In support of our authors and their future submissions to the journal, we hereby provide a brief and concise checklist based on 5 pragmatic questions to be asked before submitting a research paper [3].

## Why was the study performed?

The introduction should provide a compelling rationale for the purpose of the study. The authors must first define a relevant knowledge gap based on the existing peer-reviewed literature. Appropriate credit has to be given to previous work in the field by others. The authors should ask themselves whether the hypothesis is clinically relevant in the field of surgical patient safety. Will the answer to the study hypothesis improve to quality of clinical care delivered to surgical patients, or help resolve a previously unknown experimental problem? Perhaps the most effective method to assess the quality of the introduction is the following framework [4]: (a) state the known; (b) state the unknown (knowledge gap); (c) state the study objective (hypothesis). If a submitted manuscript lacks a defined a-priori hypothesis, the referees will likely question the overall validity of the study.

## How was the study performed?

What is the exact study design? Most surgical papers are reflective of a prospective or retrospective observational cohort study. An easy way to differentiate between prospective and retrospective studies relies on the notion that for a *prospective* study, none of the included subjects had yet developed the outcomes of interest at the time of study inception. A study design that does not meet this requirement is therefore *retrospective* by definition. Unfortunately, many submitted papers to the journal erroneously claim to report “prospective data” based on a retrospective analysis of a prospective database. Such a study design is *retrospective* by definition, since the outcomes had already occurred at the time when the study was initiated. In the field of surgery, submitted papers are frequently based on large databases because of their public availability. Unfortunately, many of these repositories are of administrative nature and consequently do not contain the elements essential to address a study hypothesis. Finally, the methods section must provide unequivocal in−/exclusion criteria for patient enrollment. The results section must match these numbers. The authors should also clarify whether the included patients were enrolled consecutively. Non-consecutive enrollment implies selection bias which limits the paper’s scientific validity overall.

Interventional studies and clinical trials are by definition *prospective* in design. The main distinguishing feature is based on the participants’ exposure to an experimental intervention assigned by the investigators, e.g. a novel medical treatment or surgical technique. In contrast, prospective cohort studies are observational and not interventional. In randomized controlled trials (RCTs), the assignment of subjects to one of the comparative treatment groups is performed by random allocation in order to mitigate the influence of confounding factors. Of note, many papers submitted to the journal claim to be reflective of a randomized trial, but do not stand the test of “true” RCT according to the CONSORT guidelines [5]. Frequent flaws in alleged randomized trials are absence of a concealed allocation modality and the lack of an intention-to-treat analysis of the data. Also, submissions that claim to represent a “surgeon-randomized” study design are not reflective of an RCT, since this designation implies that patients have been allocated to distinct surgical procedures per the surgeons’ individual expertise and personal preference, instead of random allocation.

## Are outcome measures and analytical methods appropriate?

The study design must define one single primary outcome measure that is used as the main variable to either confirm or reject the null hypothesis [6]. Frequently used outcome measures in surgical trials include in-hospital mortality, length of hospital stay, ventilator-dependent days, surgical complications, and functional or radiographic outcome scores. The primary outcome measure is used for calculating the statistical power (1-β) of the study. There can be multiple ancillary (secondary) outcome measures to support the main findings of the study. The authors have to ascertain that the selected variables of interest are suitable to test the hypothesis. Also, ask yourself if confounding factors have been taken into consideration for elimination of bias that may lead to flawed interpretation of the results. A common error is to present data as normatively distributed (mean ± SEM) rather than median ± IQR (interquartile range).

Another important aspect to take into consideration is appropriateness of the statistical analysis. Most submitted manuscripts report significant or even “highly significant” results that remain questionable if scrutinized for clinical relevance. Since the *P*-value depends on sample size, minimal differences between study groups can become statistically significant in sufficiently large sample sizes. The question is whether such negligible changes are truly clinically relevant and meaningful for patients. This problem is of increasing importance when analyzing studies that are based on large multicenter databases or national registries with hundreds of thousands of patients enrolled. The analysis of such extensive databases will make the tiniest differences in outcome parameters look statistically significant. Reciprocally, underpowered studies may not establish statistical significance despite important clinical implications, purely due to small cohort sizes (type 2 error). For this reason, it is imperative that an adequate power analysis is performed that is (a) based on the primary outcome measure and (b) capable of confirming or rejecting the null hypothesis. In general, the consultation of a professional biostatistician is recommended to ensure appropriateness of the statistical approach and power analysis.

## Are the conclusions supported by the data shown?

The discussion section of the paper should be designed to address the main question: *“How does the article I read today change what I recommend to my patients tomorrow?”* [7]. Authors are encouraged to write the discussion as a captivating “story” with a relevant conclusion and to strictly avoid the widespread pitfall of listing one published citation after the other throughout the discussion section (*“Smith et al. showed… In contrast, Jones et al. reported…”*). In general, the discussion should follow a logical sequence: (a) summary of main findings; (b) comparison to other previous publications on the topic; (c) discussion of alternative explanations for the observations; (4) clinical relevance and implications; (d) limitations of the study; (e) take-home message. Many submitted manuscripts either lack a designated conclusion section with a relevant take-home message, or the provided conclusions are not based on the scientific data shown. Any speculation and hypothetical extrapolation to aspects that have not been tested in the study should be part of the discussion, not the conclusion.

## What is the overall significance of this study?

Prior to finalizing the manuscript, the authors should ask the following questions and adjust the discussion and conclusion accordingly: (a) What are the implications of the study’s findings and conclusions? (b) Are the results novel and suitable to fill a gap in the existing published literature? (c) Can the recommendations from this study potentially justify a change in clinical practice? (d) Are the conclusions scientifically sound? (e) Are potential shortcomings and limitations of the study appropriately disclosed and addressed in the discussion? (f) Are the data clinically relevant, not just statistically significant?

In summary, the strict adherence to the proposed framework will help submitting authors to improve the scientific content and quality of presentation of their manuscripts and ultimately increase the likelihood of acceptance for publication. We would like to take the occasion to thank our authors and readers around the globe for their continuing trust and support of the journal’s mission, and for safeguarding the scientific quality of articles submitted to the journal.

## Data Availability

Please contact the author for data requests.

## References

[1] Stahel PF, Smith WR, Moore EE, Mehler PS, Weckbach S, Kim FJ, Butler N, Pape HC, Clarke TJ, Makary MA, Clavien PA (2017). The 10^th^ anniversary of patient safety in surgery. Patient Saf Surg.

[2] Patient Safety in Surgery - Instructions for authors. https://pssjournal.biomedcentral.com/submission-guidelines/preparing-your-manuscript (weblink last accessed: 12 Sept 2020).

[3] Stahel PF, Moore EE (2016). How to review a surgical paper: a guide for junior referees. BMC Med.

[4] Sauaia A, Moore EE, Crebs JL, Maier RV, Hoyt DB, Shackford SR (2014). The anatomy of an article: title, abstract, and introduction. J Trauma Acute Care Surg.

[5] CONSORT statement. www.consort-statement.org/ (weblink last accessed: 12 Sept 2020).

[6] Sauaia A, Moore EE, Crebs J, Maier R, Hoyt DB, Shackford SR (2017). The anatomy of an article: methods and results. J Trauma Acute Care Surg.

[7] Sauaia A, Moore EE, Crebs J, Maier R, Hoyt DB, Shackford SR (2013). The anatomy of an article: the discussion section – “how does the article I read today change what I will recommend to my patients tomorrow?”. J Trauma Acute Care Surg.

